# Association between pre-gravid body mass index and clinical outcomes in in vitro fertilization: a multicentered retrospective cohort study

**DOI:** 10.1186/s12884-024-06661-2

**Published:** 2024-07-09

**Authors:** Xiaoping Liu, Panyu Chen, Meng Wang, Weie Zhao, Lei Jin, Juanzi Shi, Yundong Mao, Cuilian Zhang, Xiaoyan Liang, Rui Huang

**Affiliations:** 1 Reproductive Medicine Research Center, The Sixth Affiliated Hospital, Sun Yat-sen University, Guangzhou, 510000 China; 2 Reproductive Medicine Center, The First People’s Hospital of Foshan, Foshan, China; 3 Reproductive Medicine Center, Tongji Hospital, Tongji Medicine College, Huazhong University of Science and Technology, Wuhan, China; 4 the Assisted Reproduction Center, Northwest Women’s and Children’s Hospital, Xi’an, China; 5 Reproductive Medicine Center, State Key Laboratory of Reproductive Medicine, Center of Clinical Reproductive Medicine, First Affiliated Hospital of Nanjing Medical University, Nanjing, China; 6 Reproductive Medicine Center, Henan Provincial People’s Hospital, Zhengzhou, China; 7 GuangDong Engineering Technology Research Center of Fertility Preservation, Guangzhou, China

**Keywords:** Age, Body mass index, Cumulative live birth rate, In vitro fertilization, Neonatal outcome

## Abstract

**Background:**

With the increasing incidence of obesity and the childbearing-age delay among women, a debate over obesity’s impacts on pregnancy and neonatal outcomes becomes hot. The potential negative effects of obesity and aging on fertility lead to an idea, whether an obese female pursuing IVF treatment can benefit from an ideal BMI achieved over a long-time weight loss process at the cost of aging? We aimed to assess the association between body mass index (BMI) and clinical or neonatal outcomes in patients undergoing in vitro fertilization (IVF) treatment, for answering whether it is necessary to lose weight first for obese patients, particularly those at advanced age.

**Methods:**

A retrospective cohort study was performed using multicentered data from China. The women were stratified into 5 groups in terms of pre-gravid BMI (kg/m^2^) with the WHO obesity standard (group 1: BMI < 18.5; group 2: 18.5 ≤ BMI < 23.0; group 3: 23.0 ≤ BMI < 25.0; group 4: 25.0 ≤ BMI < 30.0; group 5: BMI ≥ 30.0). The primary outcome was cumulative live birth rate (CLBR), and other clinical and neonatal outcomes were weighed as secondary outcomes. Multivariate logistic regression analyses were carried to evaluate the association between BMI and the CLBR, or between BMI and some neonatal outcomes. Furthermore, we implemented a machine-learning algorithm to predict the CLBR based on age and BMI.

**Results:**

A total of 115,287 women who underwent first IVF cycles with autologous oocytes from January 2013 to December 2017 were included in our study. The difference in the CLBR among the five groups was statistically significant (*P* < 0.001). The multivariate logistic regression analysis showed that BMI had no significant impact on the CLBR, while women’s age associated with the CLBR negatively. Further, the calculation of the CLBR in different age stratifications among the five groups revealed that the CLBR lowered with age increasing, quantitatively, it decreased by approximately 2% for each one-year increment after 35 years old, while little difference observed in the CLBR corresponding to the five groups at the same age stratification. The machine-learning algorithm derived model showed that BMI’s effect on the CLBR in each age stratification was negligible, but age’s impact on the CLBR was overwhelming. The multivariate logistic regression analysis showed that BMI did not affect preterm birth, low birth weight infant, small for gestational age (SGA) and large for gestational age (LGA), while BMI was an independent risk factor for fetal macrosomia, which was positively associated with BMI.

**Conclusions:**

Maternal pre-gravid BMI had no association with the CLBR and neonatal outcomes, except for fetal macrosomia. While the CLBR was lowered with age increasing. For the IVF-pursuing women with obesity plus advanced age, rather than losing weight first, the sooner the treatment starts, the better. A multicentered prospective study with a large size of samples is needed to confirm this conclusion in the future.

**Supplementary Information:**

The online version contains supplementary material available at 10.1186/s12884-024-06661-2.

## Background

With its worldwide prevalence in past decades, obesity, associated with metabolic, cardiovascular complications, menstrual disorders, obstetric complications and women’s infertility [1], has also become a serious public health problem in China. With China’s rapid economic progress, obesity in Chinese adults has also been soaring in the past 30 years, and, the percentage of people with BMI ≥ 24 kg/m² might reach an unprecedented 65.3% by 2030 with the absolute number approaching 800 million [2]. Simultaneously, the controversy about the impacts of obesity or overweight on pregnancy and neonatal outcomes is becoming hot in the background of the delay of first-born among modern women, which demarcates international professionals into two lines of yea or nay, the former indicating obesity’s disadvantage over clinical pregnancy rate and live birth rate [3, 4] while the latter presenting opposite data [5–7]. Similarly, no consistent conclusion can be made about the effects of obesity or overweight on the neonate or fetus [8–11].

The childbearing-age delay plus the fact that childbearing-age women gain weight with aging perplexes the clinical situation when these women refer to an IVF treatment, because the advanced age can cause adverse effects on pregnancy outcomes, and the role of obesity in women’s reproduction remains an open question. The potential negative effects of obesity and aging on fertility lead to an idea, whether an obese female pursuing IVF treatment can benefit from an ideal BMI achieved over a long-time weight loss process at the cost of aging? Recently, several prospective randomized controlled studies showed that lifestyle interventions and guidance for weight loss did not improve fertility outcomes but might increase the financial burden on patients and delay their pregnancy [12–14]. The present study aimed to assess BMI’s association with clinical and neonatal outcomes through analyses on multicentered data. It is worth noting that our BMI groups is based on the WHO obesity standard. Furthermore, based on the analyses, we tried to optimize the ART protocol for obese patients, particularly those at advanced age (≥ 35 years).

## Methods

### Study design and population

This retrospective cohort study used data from five China’s large academic reproductive medicine centers, including The Sixth Affiliated Hospital, Sun Yat-sen University, Tongji Hospital of Huazhong University of Science and Technology, Northwest Women’s and Children’s Hospital, First Affiliated Hospital of Nanjing Medical University, and Henan Provincial People’s Hospital.

### Ethical considerations

The original data collection and analysis for this study received approval from the institutional review board at each participating center, including The Sixth Affiliated Hospital, Sun Yat-sen University (2020ZSLYEC-295), Tongji Hospital of Huazhong University of Science and Technology (TJ-IRB20210320), Northwest Women’s and Children’s Hospital (2019013), First Affiliated Hospital of Nanjing Medical University (2020-SR-046), and Henan Provincial People’s Hospital (SYSZ-LL-2019110401). The five review boards waived the requirement for written informed consent from the patients because of the retrospective nature of the study.

### Data collection and extraction

The patients’ demographic, clinical, and laboratory characteristics were recorded in a digitalized, standardized recording system, particularly, their heights and weights were measured right before they started an IVF cycle. The clinical outcomes data, such as pregnancy, ectopic pregnancy, and early pregnancy loss, were collected upon the patient on-site visit, while the clinical outcomes after the 12 weeks of pregnancy as well as neonatal outcomes collected by site nurses via telephone follow-up. Throughout the present study, all the primary data from the five centers were extracted, cleaned, and standardized by a specialized data company to veil the patients’ privacy, including names and ID card numbers.

The women who underwent first IVF/ICSI cycles with autologous oocytes from January 2013 to December 2017 were subjected, and the data from fresh and all subsequent frozen-thawed embryo transfer (TET) cycles were collected. Participants were followed up until a live birth was achieved or all embryos from the same retrieval cycle had been thawed and transferred. The exclusion criteria were: (1) one of the couples had chromosomal abnormality or a genetic mutation that causes infertility; (2) sperms were of the donated or obtained surgically; (3) oocytes were of the donated or in vitro maturation; (4) multiple fertilization methods coincided in the same cycle; (5) couples underwent preimplantation genetic testing. Finally, a total of 115,287 women were included, and stratified into five groups in terms of their BMIs (kg/m^2^) with the WHO obesity standard (group 1: BMI < 18.5; group 2: 18.5 ≤ BMI < 23.0; group 3: 23.0 ≤ BMI < 25.0; group 4: 25.0 ≤ BMI < 30.0; group 5: BMI ≥ 30.0).

### Outcomes

The primary outcome was cumulative live birth rate (CLBR), which is defined as the proportion of patients who delivered at least one live birth in the fresh embryo transfer (FET) or subsequent TET cycles. The secondary outcomes included the rates of clinical pregnancy, miscarriage, live birth, twins, and neonatal outcomes including preterm birth, small for gestational age (SGA), large for gestational age (LGA), low birth weight infant and fetal macrosomia. Clinical pregnancy was defined as the presence of a gestational sac by ultrasound. Miscarriage was defined as the pregnancy loss up to the 28 weeks of gestation. Live birth was defined as the birth of at least one live infant (at least 28 weeks of gestation). Based on the Chinese birthweight reference [15, 16], SGA and LGA were defined as birthweight lower than the 10th percentile and higher than the 90th percentile of the referential birthweight, respectively.

### Statistical analyses

Continuous data are expressed as “mean (SD)” for parametric data or “median (IQR)” for non-parametric data and were compared using Student’s t test or Mann-Whitney U test as appropriate. Categorical variables are presented as numbers with percentages and were compared using Chi-square test or Fisher’s exact test. Logistic regression analyses were performed for clinical and neonatal outcomes, and multivariate logistic regression analyses performed to evaluate the association between BMI and the CLBR, or between BMI and some neonatal outcomes, with adjustments being made for co-variables and potential confounding factors. A machine-learning algorithm was implemented to predict the CLBR based on age and BMI. A sensitivity analyses was performed to check the robustness of our results. The results are presented as the odds ratio (OR) and 95% confidence interval (CI). Statistical analysis was done using R software (https://www.r-project.org/) and Python software (https://www.python.org/). All calculated *P v*alues were two sided, and a *P* value lower than 0.05 was considered statistically significant.

## Results

### Patients’ baseline and cycle characteristics

**Fig. 1:**
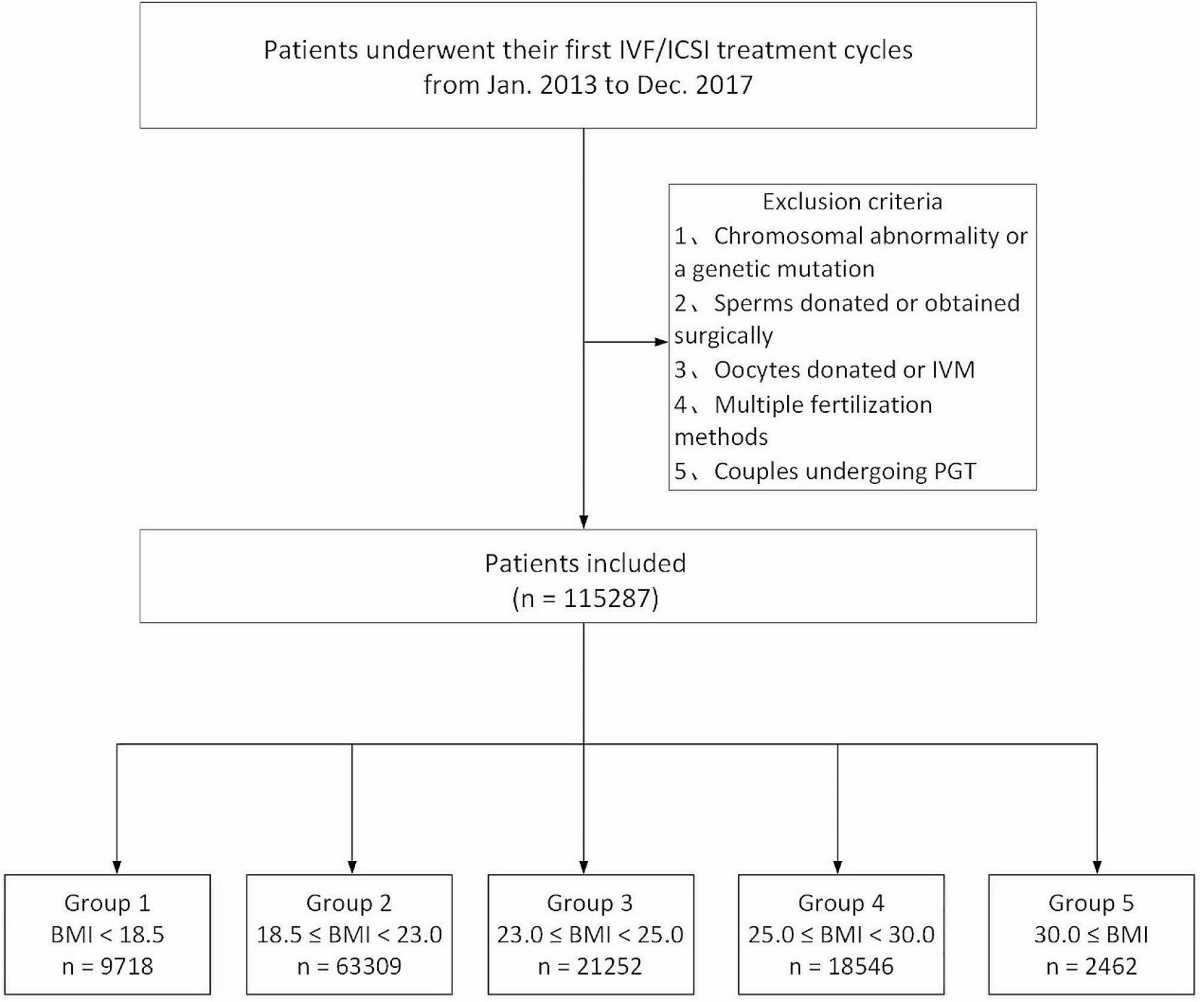
Flow chart of patient recruitment IVF = in vitro fertilization; ICSI = intracytoplasmic sperm injection; COS = controlled ovarian stimulation; IVM = in vitro maturation; PGT = preimplantation genetic testing; BMI = body mass index (kg/m^2^)

Figure 1 represents the flow chart through which 115,287 women were recruited for the study, and Table 1 lists patients’ baseline characteristics. Specifically, the baseline characteristics featured two tendencies: (1) BMI’s increase accompanied the upward proportion of PCOS patients significantly; (2) with the increasing of BMI, the proportion of young women shrank while the advanced women (> 35 years) swelled except for group 5 (BMI ≥ 30), and this trend persisted even when PCOS patients were excluded (Table SI). Cycle characteristics are listed in Table 2, showing that the differences in all the parameters were of significance among the five groups (*P* < 0.001) except the number of transferred embryos in the TET cycle.

**Table 1:** Patients’ baseline characteristics in the five groups

BMI (kg/m^2^)	Group 1 *n* = 9718	Group 2 *n* = 63,309	Group 3 *n* = 21,252	Group 4 *n* = 18,546	Group 5 *n* = 2462	*P* value
Weight (kg)	45.40 ± 3.38	53.32 ± 4.55	61.01 ± 4.23	68.02 ± 5.60	81.57 ± 8.26	< 0.001
Height (cm)	160.39 ± 5.01	159.93 ± 5.14	159.70 ± 5.27	159.44 ± 5.36	159.23 ± 6.07	< 0.001
Age (years)	29.53 ± 4.10	31.01 ± 4.74	31.91 ± 5.19	31.78 ± 5.18	30.83 ± 4.83	< 0.001
< 35	8745 (88.8%)	49,978 (78.3%)	15,220 (71.4%)	13,296 (72.1%)	1916 (79.5%)	
35–37	701 (7.1%)	7220 (11.3%)	2685 (12.6%)	2399 (13.0%)	253 (10.5%)	
38–40	291 (3.0%)	3993 (6.3%)	1839 (8.6%)	1510 (8.2%)	142 (5.9%)	
41–42	68 (0.7%)	1401 (2.2%)	829 (3.9%)	619 (3.4%)	48 (2.0%)	
≥ 43	48 (0.5%)	1237 (1.9%)	744 (3.5%)	617 (3.3%)	52 (2.2%)	
Infertility diagnosis						
Male factor	1614 (18.9%)	8625 (15.4%)	2456 (12.9%)	1954 (11.8%)	260 (11.9%)	< 0.001
PCOS	753 (8.8%)	5897 (10.5%)	2917 (15.4%)	3655 (22.1%)	733 (33.5%)	
Diminished ovarian reserve	834 (9.7%)	6679 (11.9%)	2637 (13.9%)	2050 (12.4%)	215 (9.8%)	
Tubal factor	3758 (43.9%)	24,556 (43.7%)	7901 (41.6%)	6550 (39.7%)	732 (33.5%)	
Endometriosis	829 (9.7%)	4476 (8.0%)	1042 (5.5%)	718 (4.3%)	66 (3.0%)	
Uterine factor	522 (6.1%)	4073 (7.3%)	1382 (7.3%)	1033 (6.3%)	111 (5.1%)	
Other(s)	246 (2.9%)	1861 (3.3%)	666 (3.5%)	550 (3.3%)	69 (3.2%)	
Duration of infertility (years)	3.00 (2.00, 4.58)	3.00 (2.00, 5.00)	3.00 (2.00, 5.00)	3.00 (2.00, 5.42)	4.00 (2.00, 6.00)	< 0.001
Gravidity history						
0	3781 (60.4%)	23,014 (52.2%)	7175 (46.3%)	6514 (47.3%)	1043 (54.8%)	< 0.001
≥ 1	2483 (39.6%)	21,114 (47.8%)	8326 (53.7%)	7246 (52.7%)	860 (45.2%)	
Parity history						
0	7119 (91.2%)	44,660 (84.1%)	14,351 (78.5%)	12,672 (78.9%)	1809 (84.4%)	< 0.001
≥ 1	690 (8.8%)	8454 (15.9%)	3939 (21.5%)	3385 (21.1%)	335 (15.6%)	
AMH (ng/mL)	3.93 (2.05, 6.84)	3.66 (1.91, 6.45)	3.45 (1.74, 6.50)	3.55 (1.73, 6.63)	3.48 (1.71, 6.56)	< 0.001
Basal FSH (IU/L)	7.38 (6.27, 8.77)	7.02 (5.91, 8.38)	6.74 (5.68, 8.08)	6.52 (5.48, 7.84)	6.30 (5.30, 7.50)	< 0.001
Basal LH (IU/L)	5.10 (3.80, 6.79)	4.56 (3.40, 6.10)	4.11 (3.01, 5.70)	3.83 (2.73, 5.47)	3.89 (2.58, 6.22)	< 0.001
Basal E_2_(pg/mL)	44.94 (34.00, 57.79)	40.66 (30.31, 53.10)	37.26 (27.70, 49.11)	36.00 (26.70, 47.70)	36.42 (27.00, 48.09)	< 0.001
Basal T (ng/mL)	0.34 (0.25, 0.45)	0.34 (0.25, 0.46)	0.36 (0.26, 0.48)	0.37 (0.27, 0.51)	0.39 (0.28, 0.54)	< 0.001
Antral follicle count	13.00 (9.00, 18.00)	12.00 (8.00, 18.00)	13.00 (8.00, 19.00)	14.00 (9.00, 20.00)	16.00 (10.00, 22.00)	< 0.001

Results presented as mean ± SD, frequency (percentage) or median (interquartile range)

BMI: body mass index; PCOS: polycystic ovary syndrome; AMH: anti-müllerian hormone; FSH: follicle stimulating hormone; E_2_: estradiol; LH: luteinizing hormone; T: testosterone; GnRH: gonadotropin-releasing hormone; PPOS: progestin primed ovarian stimulation; COS: controlled ovarian stimulation

Group 1: BMI < 18.5; Group 2: 18.5 ≤ BMI < 23.0; Group 3: 23.0 ≤ BMI < 25.0; Group 4: 25.0 ≤ BMI < 30.0; Group 5: BMI ≥ 30.0

**Table 2:** Patients’ cycle characteristics in the five groups

BMI (kg/m^2^)	Group 1 *n* = 9718	Group 2 *n* = 63,309	Group 3 *n* = 21,252	Group 4 *n* = 18,546	Group 5 *n* = 2462	*P* value
COS protocol						
GnRH agonist	3806 (62.3%)	24,889 (61.0%)	7500 (55.6%)	6238 (54.5%)	760 (57.0%)	< 0.001
GnRH antagonist	1471 (24.1%)	9234 (22.6%)	3202 (23.7%)	2749 (24.0%)	301 (22.6%)	
Mild ovarian stimulation	536 (8.8%)	4420 (10.8%)	1904 (14.1%)	1673 (14.6%)	190 (14.3%)	
PPOS	236 (3.9%)	1816 (4.5%)	735 (5.4%)	643 (5.6%)	73 (5.5%)	
Natural	45 (0.7%)	352 (0.9%)	127 (0.9%)	86 (0.8%)	5 (0.4%)	
COS without GnRH analogue	12 (0.2%)	97 (0.2%)	31 (0.2%)	50 (0.4%)	4 (0.3%)	
Total Gn (IU)	1800.00 (1350.00, 2475.00)	1875.00 (1350.00, 2550.00)	2025.00 (1500.00, 2650.00)	2150.00 (1575.00, 2825.00)	2550.00 (1875.00, 3300.00)	< 0.001
Duration of Gn stimulation (days)	10.00 (9.00, 11.00)	10.00 (9.00, 11.00)	10.00 (9.00, 11.00)	10.00 (9.00, 12.00)	11.00 (9.00, 13.00)	< 0.001
Serum E_2_level on trigger day (pg/mL)	3541.50 (2199.00, 5588.75)	3042.00 (1835.00, 4990.00)	2669.00 (1538.50, 4479.00)	2326.00 (1351.00, 3985.25)	2136.00 (1206.00, 3420.50)	< 0.001
Serum LH level on trigger day (U/L)	1.81 (1.08, 3.14)	1.92 (1.10, 3.46)	2.01 (1.07, 3.80)	1.87 (0.95, 3.63)	1.44 (0.71, 2.99)	< 0.001
Serum P level on trigger day (ng/mL)	1.13 (0.77, 1.83)	1.11 (0.71, 1.90)	1.06 (0.67, 1.85)	0.98 (0.61, 1.69)	0.89 (0.56, 1.37)	< 0.001
Fertilization type						
IVF	6627 (68.2%)	44,198 (69.8%)	15,028 (70.7%)	13,178 (71.1%)	1739 (70.6%)	
ICSI	3091 (31.8%)	19,111 (30.2%)	6224 (29.3%)	5368 (28.9%)	723 (29.4%)	
No. of oocytes retrieved	11.00 (7.00, 16.00)	10.00 (6.00, 15.00)	10.00 (6.00, 14.00)	9.00 (6.00, 14.00)	9.00 (6.00, 14.00)	< 0.001
No. of 2PN zygotes	7.00 (4.00, 10.00)	6.00 (3.00, 10.00)	6.00 (3.00, 9.00)	5.00 (3.00, 9.00)	5.00 (3.00, 8.00)	< 0.001
No. of Day-3 embryo having < 6 blastomeres	2.00 (1.00, 3.00)	2.00 (1.00, 3.00)	2.00 (1.00, 3.00)	2.00 (1.00, 3.00)	2.00 (1.00, 3.00)	< 0.001
No. of Day-3 embryo having 6–8 blastomeres	4.00 (2.00, 7.00)	4.00 (2.00, 6.00)	4.00 (2.00, 6.00)	4.00 (2.00, 6.00)	3.00 (2.00, 6.00)	< 0.001
No. of Day-3 embryo having > 8 blastomeres	2.00 (1.00, 3.00)	2.00 (1.00, 3.00)	2.00 (1.00, 3.00)	2.00 (1.00, 3.00)	2.00 (1.00, 3.00)	< 0.001
No. of transferrable embryos	4.00 (2.00, 6.00)	4.00 (2.00, 6.00)	3.00 (2.00, 5.00)	3.00 (2.00, 5.00)	3.00 (2.00, 5.00)	< 0.001
Rate of transferred blastocysts	33.1%	35.4%	36.3%	37.4%	40.9%	< 0.001
No. of transfer embryos in FET cycle						< 0.001
1	1995 (38.9%)	13,223 (39.1%)	4652 (40.1%)	4169 (40.6%)	612 (45.0%)	
≥ 2	3138 (61.1%)	20,557 (60.9%)	6944 (59.9%)	6088 (59.4%)	747 (55.0%)	
No. of transfer embryos in TET cycle						0.0539
1	2065 (54.6%)	14,026 (52.5%)	4774 (52.8%)	3964 (51.9%)	453 (52.4%)	
≥ 2	1642 (43.4%)	12,228 (45.7%)	4107 (45.4%)	3553 (46.5%)	399 (46.2%)	

Results presented as frequency (percentage) or median (interquartile range)

BMI: body mass index; Gn: gonadotropin; E_2_: estradiol; LH: luteinizing hormone; P: progesterone; 2PN: 2 pronuclei; FET: fresh embryo transfer; TET: frozen-thawed embryo transfer; IVF: in-vitro fertilization; ICSI: intracytoplasmic sperm injection

Group 1: BMI < 18.5; Group 2: 18.5 ≤ BMI < 23.0; Group 3: 23.0 ≤ BMI < 25.0; Group 4: 25.0 ≤ BMI < 30.0; Group 5: BMI ≥ 30.0

### Clinical outcomes

We calculated and compared the clinical outcomes among the five groups (Table 3): (1) in FET cycles: the difference in the miscarriage rate among the five groups was statistically significant (*P* < 0.001), but no statistical difference in the clinical pregnancy rate, live birth rate, and the proportions of singleton and twin births; (2) in complete cycles: the differences in the CLBR (*P* < 0.001) and in the ratio of twins (*P* = 0.0434) among the 5 groups were statistically significant.

**Table 3:** Clinical outcomes in the five groups

	BMI (kg/m^2^)	Group 1 *n* = 9718	Group 2 *n* = 63,309	Group 3 *n* = 21,252	Group 4 *n* = 18,546	Group 5 *n* = 2462	*P* value	OR (95%CI)
FET	Cycle	4577	30,411	10,474	9368	1231		
	Clinical pregnancy rate n (%)	2750 (60.08%)	18,128 (59.61%)	6303 (60.18%)	5643 (60.24%)	748 (60.76%)	0.68 ^a^	1.01 (0.99–1.03)
	Miscarriage rate n (%)	207 (7.53%)	1499 (8.27%)	621 (9.85%)	631 (11.18%)	104 (13.90%)	< 0.001 ^a^	1.18 (1.14–1.22)
	Live birth rate n (%)	2154 (47.06%)	14,179 (46.62%)	4818 (46%)	4271 (45.59%)	532 (43.22%)	0.0533 ^a^	0.97 (0.96–0.99)
	Singleton rate n (%)	1626 (75.49%)	10,854 (76.56%)	3714 (77.09%)	3277 (76.74%)	416 (78.2%)	0.57 ^b^	0.98 (0.95–1.01)
	Twins rate n (%)	528 (24.51%)	3323 (23.44%)	1104 (22.91%)	993 (23.26%)	116 (21.8%)		
FET & TET	Patient	9718	63,309	21,252	18,546	2462		
	CLBR % (n)	5769 (59.36%)	36,127 (57.06%)	11,752 (55.3%)	9945 (53.62%)	1277 (51.87%)	< 0.001 ^a^	0.93 (0.92–0.94)
	Singleton rate n (%)	4470 (77.48%)	28,258 (78.23%)	9242 (78.66%)	7820 (78.65%)	1036 (81.13%)	0.0434 ^b^	0.97 (0.95–0.99)
	Twins rate n (%)	1299 (22.52%)	7863 (21.77%)	2508 (21.34%)	2123 (21.35%)	241 (18.87%)		

Results presented as frequency (percentage)

a: Using Chi-square test; b: Using rank sum test

The OR value was obtained by logistic regression with BMI grouping as independent variable

BMI: body mass index; FET: fresh embryo transfer; TET: frozen-thawed embryo transfer; CLBR: cumulative live birth rate

The clinical outcomes in the FET were based on the number of fresh embryo transfer cycles as the denominator, and the cumulative clinical outcomes in the FET & TET were based on the number of women

Group 1: BMI < 18.5; Group 2: 18.5 ≤ BMI < 23.0; Group 3: 23.0 ≤ BMI < 25.0; Group 4: 25.0 ≤ BMI < 30.0; Group 5: BMI ≥ 30.0

Logistic analysis was applied to evaluate the effect of BMI on the CLBR (Table 4). After a univariate logistic regression analysis on the variables for initial screening (Table 5), the significant variables (*P* < 0.1) were included in the next step, a multivariate logistic regression analysis, which was performed under the consideration that the variables described in Table 5 can act as potential confounding factors. It was found that BMI had no statistically significant association with the CLBR, while women’s age associated with the CLBR negatively (Table 4). When compared with the women younger than 35 years, the CLBR in the women aged from 35 to 37 years old decreased by about 46% (OR 0.5428, 95% CI 0.4459–0.6615, *P* < 0.001), aged from 38 to 40 decreased by about 68% (OR 0.3175, 95% CI 0.2426–0.4145, *P* < 0.001), aged from 41 to 42 decreased by about 89% (OR 0.1084, 95% CI 0.0595–0.1864, *P* < 0.001), and aged ≥ 43 years old decreased by about 96% (OR 0.0386, 95% CI 0.0093–0.1082, *P* < 0.001).

**Table 4:** Multivariate logistics regression for clinical and neonatal outcome

		*P* value	OR (95%CI)
CLBR	(Intercept)	< 0.0001	0.3235(0.225–0.4647)
	Age < 35		Ref
	Age = 35–37	< 0.0001	0.5428(0.4459–0.6615)
	Age = 38–40	< 0.0001	0.3175(0.2426–0.4145)
	Age = 41–42	< 0.0001	0.1084(0.0595–0.1864)
	Age ≥ 43	< 0.0001	0.0386(0.0093–0.1082)
	No. of transferrable embryos	< 0.0001	1.2603(1.2227–1.3001)
	No. of transfer embryos	< 0.0001	2.2587(1.8881–2.7022)
Preterm birth	center	0.029	0.7858(0.6329–0.9761)
	Antral follicle	0.010	1.0510(1.0119–1.0915)
Low birth weight infant	Age	0.045	0.4225(0.1717–0.9427)
Fetal macrosomia	BMI	0.028	1.1850(1.1222–1.2513)
	No. of oocytes retrieved	0.047	1.0384(0.9470–1.1386)
	Serum E_2_ level at trigger	0.037	0.9998(0.9302–1.0745)
LGA	BMI	0.164^a^	1.0705(0.7768–1.4751)
SGA	BMI	0.432 ^a^	0.8908(0.3818–2.0782)

a: No significance

CLBR: cumulative live birth rate; BMI: body mass index; E_2_: estradiol; LGA: large for gestational age; SGA: small for gestational age

Adjusted for center, BMI group, PCOS, Therapy method, Age group, Infertility diagnosis, AMH, Antral follicle count, Total Gn, Duration of Gn stimulation, Serum E_2_ level on trigger day, No. of oocytes retrieved, No. of transferrable embryos, No. of transfer embryos

**Table 5:** Univariate logistics regression for CLBR

	*P* value	β	OR (95%CI)
center	< 0.001	0.0675	1.2060(1.1353–1.2813)
BMI group	< 0.001	-0.0753	0.9275(0.9160–0.9391)
PCOS	< 0.001	0.4715	1.6024(1.5433–1.6637)
Therapy method	< 0.001	1.4375	4.2101(3.8973–4.5479)
Age	< 0.001	-3.1284	0.0438(0.0378–0.0508)
AMH	< 0.001	0.1238	1.1317(1.1266–1.1369)
Antral follicle count	< 0.001	0.0569	1.0585(1.0566–1.0605)
Total Gn	< 0.001	-0.0002	0.9998(0.9998–0.9998)
Duration of Gn stimulation	< 0.001	0.0751	1.0781(1.0728–1.0832)
Serum E_2_ level on trigger day	< 0.001	0.0002	1.0002(1.0002–1.0002)
No. of oocytes retrieved	< 0.001	0.0622	1.0642(1.0621–1.0662)
No. of transferrable embryos	< 0.001	0.0867	1.0905(1.0847–1.0964)
No. of transfer embryos	< 0.001	0.5704	1.7689(1.7125–1.8273)

CLBR: cumulative live birth rate; BMI: body mass index; PCOS: polycystic ovary syndrome; AMH: anti-müllerian hormone; Gn: gonadotropin; E_2_: estradiol

**Fig. 2:**
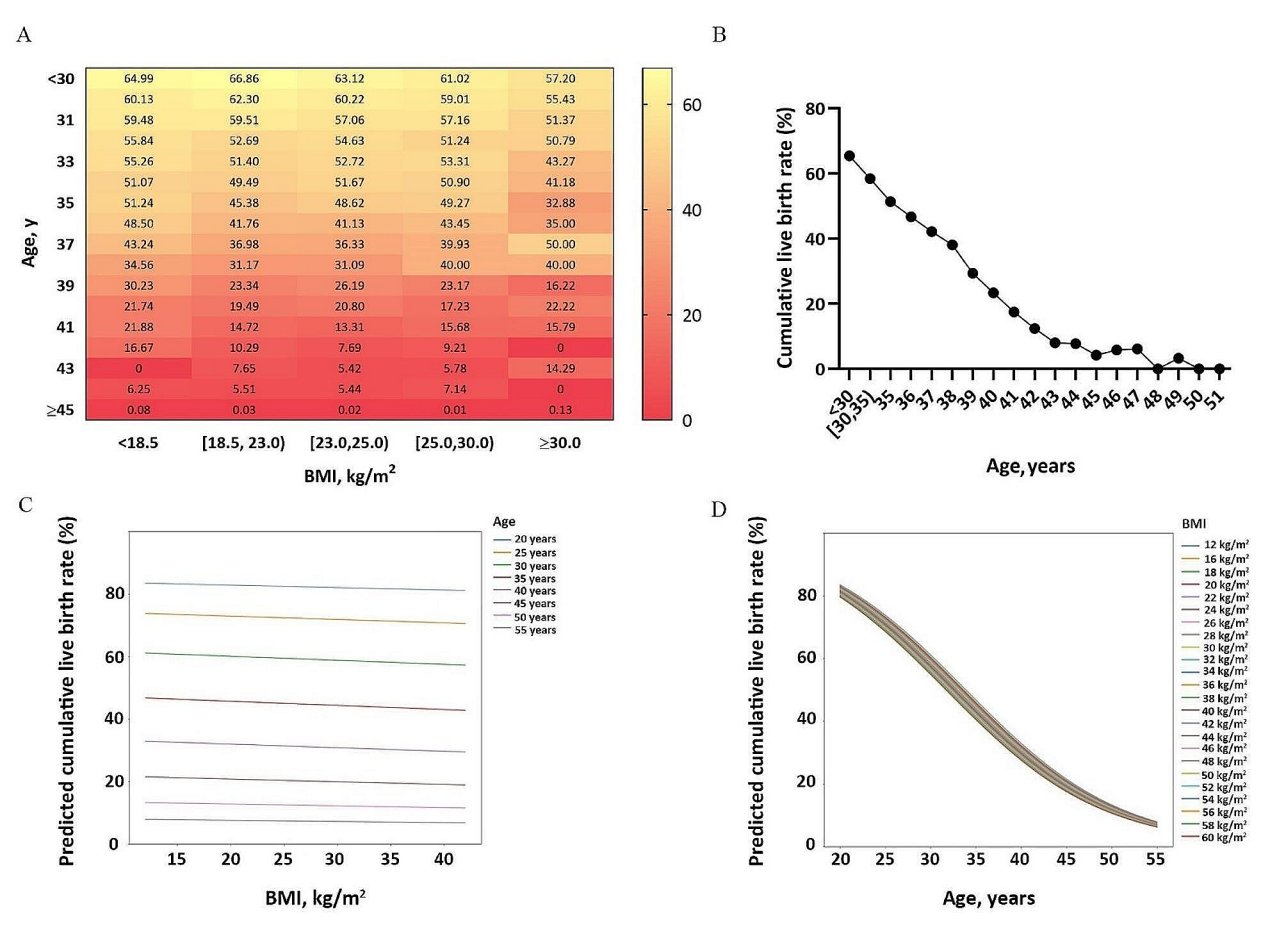
Impacts of age and BMI on CLBR. **A**: the CLBR (%) based on maternal age and BMI among 115,287 women. **B**: Line Chart of CLBR at different ages. **C**: Logistics regression model of BMI and the CLBR in different age groups. The model diagram is shaped like a straight line. In different age groups, the CLBR decreased very little as BMI increased. **D**: Logistics regression model of age and the CLBR in different BMI groups. The model diagram is shaped like adverse “S”. In different BMI groups, from the age of 20, CLBR slides down with age BMI = body mass index (kg/m^2^); CLBR = cumulative live birth rate

Furthermore, Fig. 2 refines the spectrum of the above results. In Fig. 2A, looking vertically, the CLBR lowers with the increasing of age, while, looking horizontally, little difference observed in the CLBR corresponding to the 5 groups at the same age. In Fig. 2B, evidently, the CLBR remains on a downswing with the increasing of age, quantitatively, after 35 years old, the CLBR decreased by approximately 2% for each one-year increment in age. Figure 2A and B suggest that it is age that negatively associates with the CLBR rather than BMI. The machine-learning algorithm derived predictive model showed, from different perspectives, that BMI’s effect on the CLBR in each age stratification was negligible, but age’s impact on the CLBR was overwhelming in different BMI levels (Fig. 2C and D). In our study, sensitivity analysis was conducted, and each of the five centers performed multivariate logistic regression analyses with cumulative live birth rate as the outcome. The results consistently aligned with our research findings (Table SIII).

### Neonatal outcomes

**Fig. 3:**
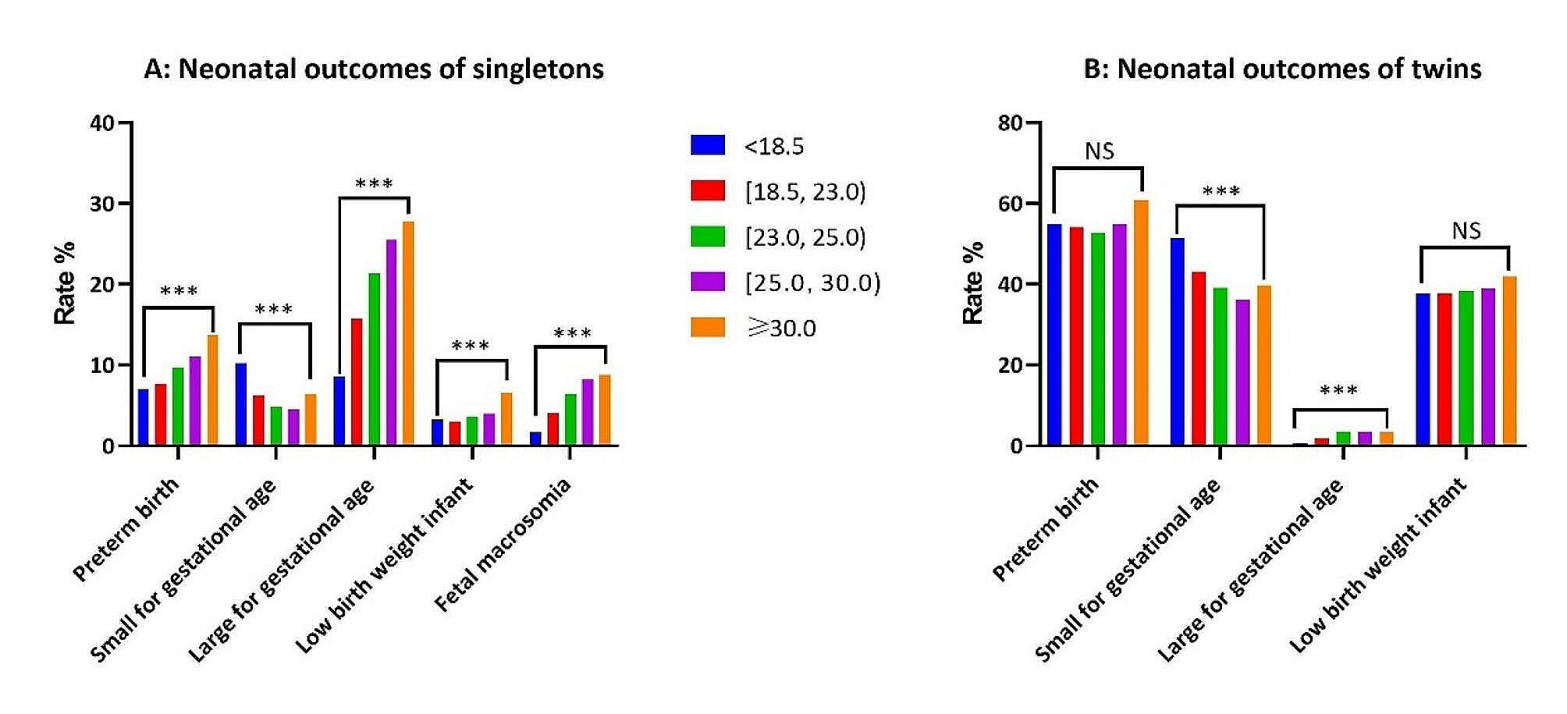
Comparison of neonatal outcomes among the five groups. **A**: Neonatal outcomes of singletons. **B**: Neonatal outcomes of twins. Blue bars = group 1, red bars = group 2, green bars = group 3, purple bars = group 4, orange bars = group 5. NS represents no significance; * *P* < 0.05; *** *P* < 0.001

Given that twin pregnancy is an influence factor in neonatal outcomes, we subdivided neonates into two: the singleton and twin groups. In the singleton group, the differences were statistically significant (*P* < 0.001) in the occurrences of preterm birth, SGA, LGA, low birth weight infant and fetal macrosomia among the 5 groups. In the twin group, the increasing of BMI was associated with a significantly lower rate of SGA (*P* < 0.001) (Fig. 3). Similarly, we performed univariate logistic regression analyses of neonatal outcomes including preterm birth, low birth weight infant, SGA, LGA and fetal macrosomia, then significant variables (*P* < 0.1) were included in the subsequent multivariate logistic regression analysis (Table 4), which showed that BMI did not affect preterm birth, low birth weight infant, SGA and LGA, and BMI was an independent risk factor for fetal macrosomia, which was positively associated with BMI (Table 4).

## Discussion

Our study included 5-centered large sample data geographically covering large areas of the south, east, north, west, and central of China, avoiding the bias from geographic deviation and applying multivariate logistic regression to adjust for confounding factors. One of the findings is that the female BMI had no association with the CLBR, but age is an independent risk factor for clinical outcomes, which is consistent with the previous literature indicating that advanced age decreases the likelihood of pregnancy [17, 18]. Noteworthily, upon the finding of no association between BMI and the CLBR, our further analyses also revealed that the CLBR decreased with the increasing of age in the five groups (Fig. 2), suggesting that for women with a high BMI, especially those at advanced age, IVF treatment should be rendered as soon as possible, instead of losing weight first.

With ART’s rapid advancement and widespread practice, mounting women are seeking IVF treatment, and the composition of patients appears to be of diversity, which make the management of the obese women who refer to IVF treatment not only a challenge but also a controversy. Several studies investigated the impact of obesity or overweight on pregnancy outcomes, some showing that obesity negatively impacted live birth rate [19, 20], while some concluding that BMI did not influence live birth rate [21, 22]. Anyway, all these studies, whether in favor of obesity’s impacts or not, only analyzed clinical outcomes after FET. Considering that TET cycles’ number has greatly increased, it is more comprehensive to assess the CLBR when evaluating obesity’s effects because the CLBR represents the clinical outcome in the entire cycle, including both FET and subsequent TET cycles. Indeed, in 2019 and 2020, two studies investigated the association between BMI and the CLBR, one showing that the CLBR was negatively impacted by increased BMI [23], and another describing an “inverted U shape” association between the two [24]. Nevertheless, distinct from the two studies, the present study found no association between BMI and the CLBR. Such discrepancy probably resulted from the following reasons. (1) Different BMI-based grouping criteria were applied. (2) Both studies only involved a single-centered small size of samples (14,782 and 15,972, respectively), but we used a multicentered large size of samples. (3) Both studies did not include the number of transferrable embryos, a very pertinent confounder, which has a significant effect on the CLBR [25]. Concretely, Xue et al. ‘s study [24] reported a lower number of retrieved oocytes in the obese group, which is consistent with our results, and it is a reasonable deduction that the true reason for the lower CLBR in their obese group was the lower number of transferrable embryos resulted from fewer retrieved oocytes, rather than BMI’s impact. In addition, they included smoking as a confounding factor, but with merely 15 (smoking) out of 14215 women, which may diverge the analysis to a biased result. The study by Zhao et al. [23] included too few confounding factors without reporting the numbers of retrieved oocytes and transferrable embryos, which, to some extent, compromises its reference value.

In addition, many studies have investigated the impact of maternal BMI on neonatal outcomes, and their results are, however, not consistent [8, 26, 27]. Therefore, we also observed obesity’s effect on neonatal outcomes in the obese women receiving IVF treatment, and the results showed that the rates of preterm birth, LGA, low birth weight infant, and fetal macrosomia in singletons increased significantly with BMI’s increasing (Fig. 2A). However, further multivariate logistics analysis by adjusting for confounding factors revealed that BMI had no association with neonatal outcomes except for fetal macrosomia (Table 4). Different from our results, a literature reported that, in FET cycles, pre-pregnancy BMI ≥ 25 kg/m^2^ was associated with a higher incidence of LGA in IVF/ICSI singletons [28], and Yang et al. reported that pre-pregnancy overweight and obesity were associated with significant increases in preterm birth, macrosomia, and LGA in TET cycles [8]. This inconsistence can be attributed to (1) the two studies were of a single-centered data research while we were a multicentered one with a large size of samples, (2) the two studies collected data of either FET or TET cycles while ours included both with more comprehensive information, (3) BMI grouping methods were different, with the WHO criteria being used by us while the couple’s BMI or Asian criteria being respectively used by them.

Obesity, an emerging global health issue affecting 603.7 million people over the past four decades [29], can impose adverse effects on reproduction, including ovulatory function, natural fecundity, and obstetric outcomes [30–32]. Parallel to the weight-increasing population, the delay of marriage and childbirth is also becoming a tendency these years [33]. Because age is a well-accepted factor relevant to pregnancy and an individual becomes fat naturally with aging, it seems that obesity and the delay of marriage and childbirth are together transforming the IVF posture, which necessitates revisiting the impact of obesity on IVF’s outcomes.

Having reviewed the pioneering research above-mentioned and bearing in mind that age is a well-accepted critical factor affecting female fertility, for finding a definitive ART treatment strategy in the obese patients at advanced age, we designed this study, which, to our knowledge, is among the only two studies to investigate the effects of BMI and age on the CLBR [34]. In contrast to our finding, reducing body weight is often recommended so far for obese women prior to infertility treatment although its effectiveness and rationality remain open to clinicians, because many guess that weight loss can bring benefits for patients based on obesity’s general adverse effects. Nevertheless, the result of a randomized controlled research argued against this doing [35], and some late clinical studies were swinging from the previous assumption that weight loss interventions improve reproductive outcomes [12, 36]. Collectively, we are here proposing readers to reconsider the decision of weight loss interventions for obese patients. Future research may determine the direct impact of weight loss in advanced women on the CLBR; however, a large and meaningful study would be challenging given the large sample size required and its prospective nature, which many subjects would not voluntarily defer long-term.

Strengths of this study include large sample size that captures data from 5 large IVF centers covering large areas of the south, east, north, west, and central of China, increasing generalizability. Our main outcome was cumulative live birth rate, which is arguably the most important outcome to patients, and was obtained using linked fresh plus frozen cycles. This study evaluated the joint impact of age and BMI. Meanwhile, this study investigated the impact of maternal BMI on neonatal outcomes.

Limitations and countermeasures are as follows. (1) This is a retrospective study in nature, and to guarantee a reliable conclusion, a large size of samples from five centers was involved, and reasonable statistical methods were applied for controlling confounding factors. (2) BMI’s variance during IVF treatment and pregnancy, which was not involved in the study, may also be a potential confounding impact on the live birth. (3) The proportion of PCOS patients was different among the five groups, and apparently there is a positive correlation between PCOS and obesity, thus introducing bias. Therefore, a multivariate regression analysis was also performed in the PCOS population, and the result showed that BMI had no effect on the CLBR in PCOS patients and the age in PCOS population was still an independent risk factor for the CLBR (Table SII). (4) BMI was the only index to define obesity without testing patients’ body fat, insulin and other indicators of lipid and glucose metabolism. (5) The outcome indicators for newborns were relatively simple, and there were no data related to maternal pregnancy complications and neonatal diseases, therefore, considering the association between obesity and other systemic diseases, maternal and neonatal safety should be investigated thoroughly in future studies.

## Conclusions

In contrast to age, an independent risk factor for the CLBR, the BMI of adult females had no association with the CLBR and neonatal outcomes, except for fetal macrosomia. For the IVF-pursuing women with obesity plus advanced age, rather than losing weight first, the sooner the treatment starts, the better. A multicentered prospective study with a large size of samples is needed to confirm this conclusion in the future.

### Electronic supplementary material

Below is the link to the electronic supplementary material.

Supplementary Material 1

## Data Availability

For study transparency and reproducibility, research data (i.e., de-identified participant data and other additional documents (i.e., statistical analysis plan) will be made available at publication upon request to the corresponding author. Interested researchers should send data access request to huangr57@mail.sysu.edu.cn. The corresponding author will review the requests with other authors for consideration. Data sharing will only be available for academic research, instead of other objectives (e.g., commercial use). A data use agreement will be required before the release of participant data and institutional review board approval as appropriate.

## Abbreviations

ART: Assisted reproductive technology
BMI: Body mass index
CLBR: Cumulative live birth rate
FET: Fresh embryo transfer
ICSI: Intracytoplasmic sperm injection
IVF: In vitro fertilization
LGA: Large for gestational age
SGA: Small for gestational age
TET: Frozen-thawed embryo transfer
