# Supplementary material for: Association between pre-gravid body mass index and clinical outcomes in in vitro fertilization: a multicentered retrospective cohort study
Source: BMC Pregnancy Childbirth. 2024 Jul 9;24:469. doi: 10.1186/s12884-024-06661-2 (PMC11234549; doi:10.1186/s12884-024-06661-2)
Supplement: Supplementary file 1 — Supplementary Material 1 [file 12884_2024_6661_MOESM1_ESM.doc]

Table SI Age of patients without PCOS in the five groups

| BMI (kg/m^2^) | Group 1 | Group 2 | Group 3 | Group 4 | Group 5 | *P* value |
| --- | --- | --- | --- | --- | --- | --- |
| **age (years)** | 29.67 ±4.21 | 31.28 ±4.86 | 32.41 ±5.32 | 32.48 ±5.34 | 31.60 ±5.19 | <0.001 |

Results presented as mean ± SD.

Table SII Multivariate logistics regression for CLBR in PCOS patients

|  | Estimate | Std.Error | z value | *P* value(>|z|) | OR (95%CI) |
| --- | --- | --- | --- | --- | --- |
| (Intercept) | -1.8005 | 0.5619 | -3.2050 | 0.0014 | 0.1652(0.0542,0.4927) |
| Age < 35 |  |  |  |  | Ref |
| Age = 35-37 | -0.7480 | 0.2831 | -2.6420 | 0.0082 | 0.4733(0.2722,0.8294) |
| Age = 38-40 | -1.0665 | 0.3897 | -2.7370 | 0.0062 | 0.3442(0.1583,0.7384) |
| Age = 41-42 | -2.1145 | 0.8252 | -2.5620 | 0.0104 | 0.1207(0.0174,0.5243) |
| Duration of Gn stimulation | 0.0915 | 0.0320 | 2.8620 | 0.0042 | 1.0958(1.0306,1.1684) |
| No. of transferrable embryos | 0.1889 | 0.0309 | 6.1110 | <0.0001 | 1.2079(1.1388,1.2857) |
| No. of transfer embryos | 0.7808 | 0.2129 | 3.6680 | 0.0002 | 2.1832(1.4361,3.3133) |

CLBR: cumulative live birth rate; PCOS: polycystic ovary syndrome; Gn: gonadotropin.

Adjusted for center, BMI group, Therapy method, Age group, AMH, Antral follicle count, Total Gn, Duration of Gn stimulation, Serum E_2_ level on trigger day, No. of oocytes retrieved, No. of transferrable embryos, No. of transfer embryos.

Table SIII Multivariate logistics regression for CLBR at five reproductive centers

|  | Characteristic | OR*^1^* | 95% CI*^1^* | p-value |  |
| --- | --- | --- | --- | --- | --- |
| Center 1  (Tongji Hospital of Huazhong University of Science and Technology) | Age |  |  | <0.001 |  |
|  | Age < 35 | — | — |  |  |
|  | Age = 35-37 | 0.41 | (0.28, 0.59) |  |  |
|  | Age = 38-40 | 0.23 | (0.14, 0.37) |  |  |
|  | Age = 41-42 | 0.09 | (0.04, 0.20) |  |  |
|  | Age ≥ 43 | 0.04 | (0.01, 0.15) |  |  |
|  | No. of transferrable embryos | 1.28 | (1.15, 1.43) | <0.001 |  |
|  | No. of transfer embryos | 2.75 | (1.97, 3.85) | <0.001 |  |
| Center2  (Henan Provincial People's Hospital) | Age |  |  | <0.001 |  |
|  | Age < 35 | — | — |  |  |
|  | Age = 35-37 | 0.66 | (0.54, 0.82) |  |  |
|  | Age = 38-40 | 0.37 | (0.28, 0.49) |  |  |
|  | Age = 41-42 | 0.16 | (0.08, 0.29) |  |  |
|  | Age ≥ 43 | 0.08 | (0.02, 0.20) |  |  |
|  | Antral follicle count | 1.02 | (1.01, 1.04) | 0.005 |  |
|  | No. of oocytes retrieved | 1.07 | (1.03, 1.10) | <0.001 |  |
|  | No. of transferrable embryos | 1.31 | (1.23, 1.39) | <0.001 |  |
|  | No. of transfer embryos | 1.54 | (1.33, 1.77) | <0.001 |  |
| Center3  (First Affiliated Hospital of Nanjing Medical University) | Age |  |  | <0.001 |  |
|  | Age < 35 | — | — |  |  |
|  | Age = 35-37 | 0.65 | (0.50, 0.83) |  |  |
|  | Age = 38-40 | 0.41 | (0.29, 0.58) |  |  |
|  | Age = 41-42 | 0.14 | (0.06, 0.32) |  |  |
|  | Age ≥ 43 | 0.04 | (0.00, 0.19) |  |  |
|  | No. of transferrable embryos | 1.28 | (1.23, 1.33) | <0.001 |  |
|  | No. of transfer embryos | 2.02 | (1.62, 2.51) | <0.001 |  |
| Center4  (Northwest Women’s and Children's Hospital) | Age |  |  | <0.001 |  |
|  | Age < 35 | — | — |  |  |
|  | Age = 35-37 | 0.65 | (0.45, 0.92) |  |  |
|  | Age = 38-40 | 0.38 | (0.24, 0.57) |  |  |
|  | Age = 41-42 | 0.22 | (0.08, 0.51) |  |  |
|  | Age ≥ 43 | 0.05 | (0.01, 0.19) |  |  |
|  | No. of oocytes retrieved | 1.06 | (1.02, 1.11) | 0.005 |  |
|  | No. of transferrable embryos | 0.93 | (0.87, 0.99) | 0.018 |  |
|  | No. of transfer embryos | 1.51 | (1.17, 1.95) | 0.001 |  |
| Center5  (The Sixth Affiliated Hospital of Sun Yat-sen University) | Age |  |  | <0.001 |  |
|  | Age < 35 | — | — |  |  |
|  | Age = 35-37 | 0.68 | (0.59, 0.78) |  |  |
|  | Age = 38-40 | 0.47 | (0.39, 0.56) |  |  |
|  | Age = 41-42 | 0.18 | (0.13, 0.26) |  |  |
|  | Age ≥ 43 | 0.06 | (0.03, 0.12) |  |  |
|  | AMH | 1.04 | (1.01, 1.07) | 0.008 |  |
|  | Total Gn | 1.00 | (1.00, 1.00) | <0.001 |  |
|  | Duration of Gn stimulation | 1.07 | (1.03, 1.01) | 0.001 |  |
|  | Serum E_2_ level on trigger day | 1.00 | (1.00, 1.00) | <0.001 |  |
|  | No. of transfer embryos | 2.75 | (2.43, 3.12) | <0.001 |  |
| *^1^* OR = Odds Ratio, CI = Confidence Interval | | | | |  |
| CLBR: cumulative live birth rate; PCOS: polycystic ovary syndrome; Gn: gonadotropin; AMH: anti-müllerian hormone  Adjusted for BMI group, PCOS, Therapy method, Age group, AMH, Antral follicle count, Total Gn, Duration of Gn stimulation, Serum E_2_ level on trigger day, No. of oocytes retrieved, No. of transferrable embryos, No. of transfer embryos. | | | | |  |
